# Aberrant Brain Activity at Early Delay Stage Post-radiotherapy as a Biomarker for Predicting Neurocognitive Dysfunction Late-Delayed in Patients With Nasopharyngeal Carcinoma

**DOI:** 10.3389/fneur.2019.00752

**Published:** 2019-07-16

**Authors:** Yadi Yang, Xiaoshan Lin, Jing Li, Lujun Han, Zhipeng Li, Shiliang Liu, Gangqiang Hou, Chuanmiao Xie, Xiaofei Lv, Yingwei Qiu

**Affiliations:** ^1^Department of Medical Imaging, Sun Yat-sen University Cancer Center, State Key Laboratory of Oncology in South China, Collaborative Innovation Center for Cancer Medicine, Guangdong Key Laboratory of Nasopharyngeal Carcinoma Diagnosis and Therapy, Guangzhou, China; ^2^Department of Radiology, The Third Affiliated Hospital of Guangzhou Medical University, Guangzhou Medical University, Guangzhou, China; ^3^Department of Radiation Oncology, Sun Yat-sen University Cancer Center, State Key Laboratory of Oncology in South China, Collaborative Innovation Center for Cancer Medicine, Guangdong Key Laboratory of Nasopharyngeal Carcinoma Diagnosis and Therapy, Guangzhou, China; ^4^Shenzhen Kangning Hospital, Shenzhen Mental Health Center, Shenzhen, China

**Keywords:** radiotherapy, radiation-induced injury, regional homogeneity, resting-state fMRI, nasopharyngeal carcinoma, neurocognitive dysfunction

## Abstract

**Background:** Increasing evidence indicates that early radiation-induced subtle cerebral changes may be the precursors to permanent brain dysfunction at the late-delayed (LDS) post-radiotherapy (RT) stage. In this study, we aim to track the RT-related longitudinal brain activity in nasopharyngeal carcinoma (NPC) patients and to determine whether early abnormal brain activity can predict late neurocognitive dysfunction after RT.

**Methods:** Thirty-three NPC patients were finally included and longitudinally followed up at the following time points: prior to treatment initiation, early-delayed stage (EDS, 1–3 months), and LDS (six months) after RT. Fifteen comparable healthy controls (HCs) were finally included and followed up in parallel. Montreal Cognitive Assessment (MoCA) was used to assess the general cognitive function. Brain activity was recorded via resting-state fMRI and regional homogeneity (ReHo). A whole-brain voxel-wise-based one-way repeated-measure analysis of variance (ANOVA) was conducted to evaluate the longitudinal ReHo changes among the three time points for NPC patients and HCs, respectively. Results were reported at the significant level of a threshold of two-tailed voxel-wise *P* < 0.01 and cluster level *P* < 0.05 with Gaussian Random Field (GRF) correction. Finally, the efficacies of the aberrant ReHo at EDS for predicting the cognitive impairment at LDS in NPC patients were evaluated.

**Results:** Significant differences were detected in ReHo among the three time points in NPC patients but not in HCs. Aberrant ReHo was distributed in the bilateral cerebellum, the right temporal lobe, and the left insular areas, which showed different dynamic changes patterns over time. Logistic regression model combining the mean ReHo, age, and irradiation dose on the bilateral temporal lobe had the highest diagnostic efficiency according to the area under the curve (AUC) score (AUC = 0.752, *P* = 0.023).

**Conclusions:** The post-RT brain activity revealed by ReHo in NPC patients was dynamic, complex, and multifactorial. Furthermore, the combination of the aberrant ReHo at EDS, age, and irradiation dose may serve as a potential biomarker of the RT-induced cognitive impairments at LDS.

## Introduction

There were an estimated 129,000 new cases of nasopharyngeal carcinoma (NPC) and 73,000 deaths in 2018 (1). According to world area, incidence rates are highest in South-Eastern Asia, including Malaysia, Indonesia, Singapore, and South-Eastern China (2). The standard treatment for NPC is radiotherapy (RT) due to the radio-sensitivity of the tumor (3). The survival rate of NPC patients is generally favorable, thus long-term side effects of RT are of concern in survivors. Radiation-induced brain injury is a major neurologic complication following RT. Classically, it is classified into an acute stage (days to weeks post-irradiation), early-delayed stage (EDS) (1–4 months post-irradiation), and late-delayed stage (LDS) (more than 4–6 months post-irradiation) (4). Radiation-related neurocognitive decline, which may be attributed to the brain injury, is a significant but largely unrecognized sequela that appears after irradiation in NPC patients (4, 5). Reportedly, it has been described as part of a biphasic pattern of cognitive loss. Following RT there is an initial deterioration in cognitive function, after which there is a transient recovery at post-RT EDS, and then a progressive, irreversible deterioration in the cognitive functioning at post-RT LDS (6). Historically, the primary research focus has been placed on markers of brain damage and cognitive decline appearing over 6 months to 1 year or later after irradiation (7). However, such knowledge cannot ameliorate the progression of the brain injury and cognition dysfunction, which is irreversible at post-RT LDS (6). Recent evidence indicates that early radiation-induced subtle changes in the brain may be the precursors to long-term, permanent brain dysfunction at post-RT LDS (4, 7, 8). Thus, consideration of early forms of RT-induced brain damage and how they can evolve over time may not only shed light on the complicated pathogenesis of RT-induced neurocognitive impairment, but also facilitate the early identification and treatment, which can reverse the degenerative processes before they have caused permanent disability.

Earlier research has suggested that brain activity in the resting-state reflects the baseline status of the whole brain and is a promising indicator in the investigation of the pathophysiological characteristics of nervous system diseases (9). Recently, resting-state brain activity was used to explore the post-RT brain dysfunction in NPC patients, which yielded inspiring findings (10–13). Two cross-sectional studies (11, 14), comparing between NPC patients who had already finished RT with those who had not received RT, revealed RT-related aberrant whole-brain and cerebellar-cerebral functional connectivity (FC), respectively. Another cross-sectional study (13), compared pre- and post-RT NPC individuals with multiple groups of different time points, which discovered that the brain activity underwent dynamic post-RT changes. Furthermore, the increased local brain activity in the inferior temporal lobe at EDS may be used to predict the occurrence of severe brain necrosis many years later (13). However, it is difficult to obtain a clear picture from these cross-sectional results, given that the post-RT cohort effects and diverse time points in different studies (11, 13). Extant longitudinal studies revealed that disruption of the FC of the hippocampal-related cortices occurred in the NPC patients (10) or intra- and inter-network disconnection within a few months after RT (12). Unfortunately, these longitudinal studies did not involve cognitive tests (10) or focused only on one post-RT time point (12), which hampered the determination of the relation of the dynamic alteration to the cognitive dysfunction. Furthermore, whether the early brain activity changes can be used for predicting late-delayed cognitive dysfunction in NPC patients is still debatable.

Regional homogeneity (ReHo) is an important research method for mapping the level of local activity across the whole brain of an individual, which reflects the local temporal homogeneity of the regional blood oxygen level-dependent signal in the resting state (15, 16). This data-driven method provides analysis of the region-to-region interactions and voxel-by-voxel neural activity with remarkably high test-retest reliabilities at the functional level, based on the intrinsic activity of the resting brain (16). Accumulating evidence indicates that the regional properties of the intrinsic brain dynamics can reliably reflect aspects of cognitive function (17–19). Furthermore, increasingly more studies have suggested the potential of ReHo changes as a prognostic imaging tool to identify the disease-related progression treatment response and outcomes for various neuropsychiatric disorders (19–21). Thus, the application of this method might be helpful for the better characterization of the relationship between the functional evolutional processes and the late-delayed cognitive dysfunction in post-RT NPC.

Given that the previous evidence of a dynamic brain activity alteration pattern during the different post-RT phases and early increased local brain functional activity was predictive of severe later temporal lobe necrosis (13), we hypothesized that: (1) Compared with pre-RT, aberrant ReHo can be detected in brains of post-RT NPC patients at EDS and LDS, which would undergo a dynamic alteration from EDS to LDS; (2) The global cognitive function would be impaired in post-RT NPC patients; (3) The abnormal ReHo index at EDS may be used as a biomarker for prediction of impaired cognitive function at LDS.

## Materials and Methods

### Participants

This prospective study was approved by the local institutional review board. Written informed consent was obtained from all subjects. From December 2014 to May 2018, 38 newly diagnosed, treatment-naive patients with NPC and 20 comparable healthy controls were initially included. Ten subjects (5 NPC patients and 5 healthy controls) were discarded due to excessive head motion. Finally, 33 patients with NPC (21 male and 12 female, 18–55 years old, mean age of 38.91 ± 9.38 years) and 15 comparable healthy controls (10 males and 5 females; aged 26–55 years, mean age 40.33 ± 10.33 years) were included. The following inclusion criteria were used for all participants: aged 18–60 years, dextromanuality, no intracranial invasion, no distant metastases, no brain tumors, no alcoholism, no substance dependence, no prior substantial head trauma, no diabetes, no viral hepatitis, no positive human immunodeficiency virus status, no neurological or psychiatric diseases or other major medical issues, routine brain MR examination was negative, and baseline MoCA scores were more than or equal to 26. The exclusion criteria for all participants were as follows: age lower than 18 or above 60 years, left-handedness, alcoholism, diabetes, brain tumors, history of cranial trauma, history of any psychiatric or neurological disease, any current medications that may affect cognitive function, contraindications for MRI scanning, and excessive head movement during the functional MRI (fMRI) acquisition. The additional exclusion employed criteria for NPC patients were intracranial invasion and distant metastases. Each NPC patient underwent a detailed pre-treatment evaluation, and the clinical stages of NPC were classified according to the 7th edition of the American Joint Committee (AJCC) staging system (22).

### Treatment

All patients received one fraction of intensity-modulated radiation therapy (IMRT) (*n* = 30) or tomotherapy (*n* = 3) daily for five consecutive days per week. The prescribed radiation doses for patients treated were 62–70 Gy at 2.0–2.33 Gy/fraction over 30–33 fractions to the planning target volume (PTV) of the nasopharynx tumor volume (GTVnx) and gross tumor volume of malignant lymph nodes (GTVnd), with 56–60 Gy to the PTV of clinical target volume 1 (CTV1) (high-risk regions) and 50–56 Gy to the PTV of CTV2 (low-risk regions and neck nodal regions). The details of the RT techniques were identical to those reported in previous studies (23, 24). Dose evaluation was performed based on the data from the dose–volume histogram for the targets (24). The main evaluation parameters were maximum dose (Dmax), mean dose (Dmean), and minimum dose (Dmin) received by bilateral temporal lobe (Table 1). In addition, of the 33 patients, 1 (3.0%) underwent only RT, 16 (48.5%) were administered concurrent chemoradiotherapy, and 16 (48.5%) received a combination of neoadjuvant and concurrent chemoradiotherapy. Neoadjuvant therapy consisted of cisplatin with 5-fluorouracil (PF), cisplatin with docetaxel (TP), or cisplatin with 5-fluorouracil and docetaxel (TPF) every 3 weeks for ≥2 cycles. The concurrent chemotherapy consisted of cisplatin/nedaplatin or paclitaxel administered weekly for at least 4–7 cycles or in weeks 1, 4, and 7 of radiation therapy.

**Table 1:** Dose–volume statistics of the bilateral temporal lobe for 33 patients with nasopharyngeal carcinoma treated with radiation therapy (Gy).

**Temporal lobe**	**Maximum dose (Dmax)**	**Minimum dose (Dmin)**	**Mean dose (Dmean)**
Left	65.51 ± 12.36	1.82 ± 0.78	17.97 ± 5.35
Right	67.59 ± 6.46	1.77 ± 0.66	18.02 ± 4.71

*All data are presented as mean ± standard deviation*.

### Image Acquisition

All MRI scans were performed on a GE Discovery MR750 3.0 scanner (GE Medical Systems, Milwaukee, WI, USA) with a 16-channel head and neck coil (GE Medical Systems) at the Department of Medical Imaging, Sun Yat-sen University Cancer Center. To detect intracranial lesions, routine imaging examinations were performed of axial T1-weighted images [repetition time (TR)/echo time (TE) = 596/8 ms] and T2-weighted images (TR/TE = 3,223/89 ms) and T2-FLAIR images [TR/TE/inversion time (TI) = 9,000/93/2,475 ms], obtained for every subject. Then, a resting-state fMRI scan with an echo-planar imaging sequence and a high-resolution structural MRI scan with T1 weighted three-dimensional brain volume imaging (3D-BRAVO) sequence were sequentially conducted. The imaging parameters were as follows: (1) Resting-state fMRI: TR/TE = 2,000/30ms, flip angle = 90°, thickness/gap = 3/0.8 mm, acquisition matrix = 64 × 64, field of view (FOV) = 240 × 240 mm^2^, voxel size = 3.75 × 3.75 × 3.8 mm^3^, 39 axial slices and 240 time points (8 min). (2) 3D-BRAVO: TR/TE = 8.16/3.18 ms, inversion time = 800 ms, flip angle = 8°, acquisition matrix = 256 × 256, FOV = 256 × 256 mm^2^, voxel size = 1 × 1 × 1 mm^3^, 176 sagittal slices with no inter-slice gap. For the resting-state fMRI scan, subjects were instructed to avoid falling asleep, keep their eyes closed, and avoid thinking about anything.

### Neurocognitive Tests

General cognitive function was assessed through the Montreal Cognitive Assessment (MoCA, Beijing Version) test, which assesses different cognitive domains: attention and concentration, executive functions, memory, language, visuo-constructional skills, conceptual thinking, calculations, and orientation. The MoCA was reported to be a feasible and relative sensitive instrument for routine cognitive screening for NPC patients with radiation-induced injury (25). Cutoff scores for the MoCA was determined at 26 (scores of 25 or below will indicate cognitive impairment) (26). The time to administer the MoCA was approximately 10 min. The MoCA scores ranged from 0 to 30; higher scores indicated a better cognitive performance. After an appropriate explanation, all subjects completed the MoCA test on the same day of MRI scanning.

### Follow-Up Procedure

**Figure 1:**
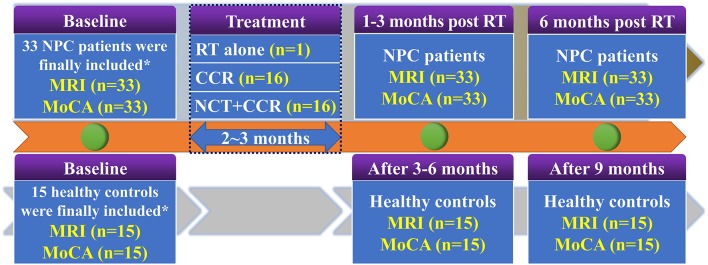
Enrolment and follow-up procedures for patients with NPC and healthy controls. CCR, Concurrent Chemotherapy and Radiotherapy; MoCA, Montreal Cognitive Assessment; NCT, Neoadjuvant Chemotherapy; NPC, nasopharyngeal carcinoma; RT, radiation therapy. ^*^38 newly diagnosed patients with NPC and 20 comparable healthy controls were initially included. Ten subjects (5 NPC patients and 5 healthy controls) were discarded due to excessive head motion. Finally, 33 patients with NPC and 15 comparable healthy controls were included.

To investigate the dynamic alteration of whole-brain ReHo and cognitive function dysfunction at an early stage after RT, all 33 patients with NPC were longitudinally evaluated at three time points: prior to treatment initiation, EDS (1–3 months after RT completion), and LDS (6 months after the completion of RT). MRI data and MoCA tests were acquired at each time point. Meanwhile, all 15 healthy controls were followed up in parallel (baseline, 6 months, 9 months) and completed MRI scans and MoCA measurements were conducted at each time point. The procedures for the follow-up of all participants are presented in Figure 1.

### Data Preprocessing and Computation of ReHo Maps

All preprocessing steps were carried out using the toolkit of Data Processing & Analysis of Brain Imaging (DPABI_V3.0_171210, http://rfmri.org/DPABI), an extension of Statistical Parametric Mapping (SPM8) (http://www.fil.ion.ucl.ac.uk/spm). The first 10 volumes of each functional time series were discarded from analysis to allow for magnetization equilibrium and for the adaptation of the subjects to the scanning situation. The remaining 230 volumes were corrected for the acquisition time delay between the different slices as well as for geometrical displacements according to the estimated head movement and were then realigned to the first volume. Head motion parameters were computed by estimating the translation in each direction and the angular rotation on each axis for each volume. Any subject who had a maximum displacement in any of the three cardinal directions (x, y, z) > 2.0 mm or a maximum spin (x, y, z) > 2.0° was excluded from the study. The realigned fMRI data were spatially normalized to the Montreal neurological institute (MNI) space using the normalization parameters estimated by T1 structural image unified segmentation and was resampled to 3 × 3 × 3 mm^3^ voxels. Several sources of spurious variances, including the estimated motion parameters, the linear drift, and the average time series in the cerebrospinal fluid and white matter regions, were removed from the data through linear regression. After that, a temporal filter (0.01–0.08 Hz) was performed to reduce the effect of low-frequency drift sand high-frequency uninteresting signals.

The calculation procedure of ReHo maps calculation was the same as that reported earlier (17, 18, 21). In brief, this was accomplished on a voxel-by-voxel basis by calculating Kendall coefficient of concordance (KCC) of time series of a given voxel with those of its nearest 26 neighbors. A larger value for a given voxel indicated a higher regional homogeneity within a cluster made up of the voxel and its nearest neighbors. A whole-brain map of ReHo values for each subject was calculated. Then, each ReHo map was scaled by its global mean, and finally smoothed with a 6 mm full-width at half maximum (FWHM) Gaussian kernel.

### Statistical Analysis

#### Statistical Analysis of the NPC Patients and Healthy Controls at the Baseline

Two-sample *t*-test was applied to compare the group difference in age, education level and MOCA between NPC patients and healthy controls at baseline. Pearson's chi-squared test was used to evaluate gender differences between the two groups. Analyses were conducted using SPSS 18.0 (SPSS for Windows, Chicago, IL, USA), and a *P* < 0.05 was deemed significant.

To investigate differences in ReHo between the two groups at baseline, a two-sample *t*-test was executed on the individual ReHo maps in a voxel-by-voxel manner using age, gender, and years of formal education as covariates.

#### Longitudinal Changes in Cognitive Function

Repeat measurement ANOVA and multiple comparisons (*post-hoc* Dunnett's tests) were employed to investigate the difference between the MOCA score among pre-RT, EDS and LDS in patients with NPC and healthy controls. Cognitive function impairment was defined if the MoCA score is <26 during the follow-up according to previous studies.

#### Longitudinal Changes of ReHo

To explore the longitudinal ReHo differences among pre-RT, EDS and LDS in patients with NPC. A whole-brain voxel-wise-based one-way repeated-measure analysis of variance (ANOVA), with head motion parameters as covariates, was conducted to evaluate the ReHo changes among the three time points. Results were reported at the significant level of a threshold of two-tailed voxel-wise *P* < 0.01 and cluster level *P* < 0.05 with Gaussian Random Field (GRF) correction. We then extracted the mean ReHo values that showed significant differences among the three time points for all individuals. One-way repeated measure ANOVA *post-hoc* Dunnett's tests with were performed to detect the dynamic alteration. Also, to exclude the aging effects, we also investigated the longitudinal ReHo differences for the normal controls using the same statistical methods.

#### Prediction of Impaired Cognitive Function in LDS Using the ReHo Index in EDS

To explore whether the ReHo alterations at EDS can be used to predict the cognitive impairment at LDS in patients with NPC, multivariate logistic regression and receiver operating characteristic curve (ROC) analyses were performed to determine the efficacies of the average ReHo value within all the clusters that showed significant changes among three time points at EDS and the clinical characteristics (age and the maximum irradiation dose of bilateral temporal lobe) alone or combined to predict impaired cognitive function at LDS. A *P-*value was considered significant if it was 0.05 or less at a confidence interval of 95%.

## Results

### Demographic Characteristics and the Comparison Results of ReHo at Baseline

The demographic and clinical characteristics of NPC patients and healthy controls at the baseline are summarized in Table 2. No significant differences were found in age, gender, education level, and MoCA scores between the two groups at the baseline. Additionally, no significant difference was detected in the whole-brain ReHo between the two groups at the baseline.

**Table 2:** Demographic and clinical characteristics of the patients with nasopharyngeal carcinoma (NPC) and healthy controls at the baseline.

**Demographic information**	**NPC patients**	**Healthy controls**	**t/χ^2^ values**	***P-*values**
Numbers	33	15	NA	NA
Age (years)^*^	39.91 ± 9.38	40.33 ± 10.33	−0.473	0.639
Gender (male/female)	21/12	10/5	0.041	0.839
Education (years)	12.42 ± 2.98	12.80 ± 3.36	−0.389	0.699
MoCA	29.18 ± 1.16	28.73 ± 0.96	1.307	0.198
T-classification (T1/T2/T3/T4)	2/5/15/11	NA	NA	NA
N-classification (N0/N1/N2/N3)	5/11/13/4	NA	NA	NA
M-classification (M0/M1)	33/0	NA	NA	NA
AJCC TMN stage (I/II/III/IV)	0/4/14/15	NA	NA	NA
RT technology (IMRT/TOMO)	30/3	NA	NA	NA
Therapeutic regimens (RT alone/CCR/NCT+CCR)	1/16/16	NA	NA	NA

* *Data are mean ± standard deviation. AJCC, American Joint Committee on Cancer; IMRT, intensity-modulated radiation therapy; MoCA, Montreal Cognitive Assessment; NA, not available; RT, radiotherapy; TOMO, tomotherapy; T-classification describes the size of the primary tumor and whether it has invaded nearby tissue. N-classification describes nearby lymph nodes that are involved. M-classification defines distant metastasis. AJCC TMN stage was obtained based on the T-, N-, and M-classification results. Therapeutic regimens include radiotherapy alone (RT alone), concurrent chemoradiotherapy (CCR) and Neoadjuvant/adjuvant chemotherapy combined with concurrent chemoradiotherapy (NCT+CCR)*.

### Longitudinal Changes in the Cognitive Function

**Figure 2:**
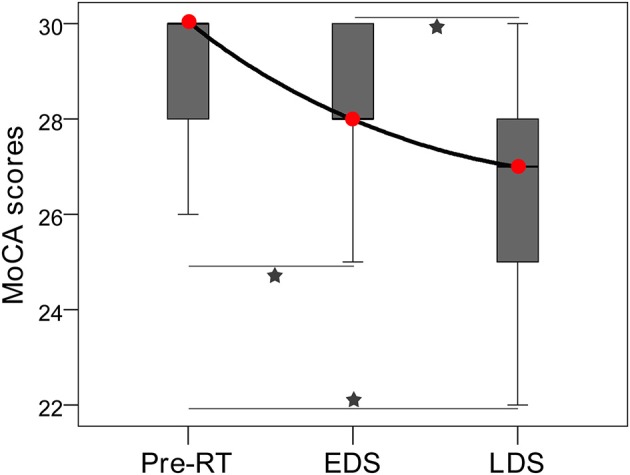
Box plot showing the longitudinal changes in MoCA performance of patients with nasopharyngeal carcinoma. A significant decrease in the MoCA scores was detected among the three stages; there were significant differences in MoCA scores between pre-RT and EDS, between Pre-RT and LDS, and between EDS and LDS. Red circles indicate median MoCA scores of each stages. Pentagrams indicate significant differences with *post-hoc* Dunnett's tests (*P* < 0.05). EDS, early-delayed stage; LDS, late-delayed stage; MoCA, Montreal Cognitive Assessment; RT, radiation therapy.

In total, 99 and 45 MoCA data were collected for NPC patients and healthy controls, respectively. A significant decrease in the MoCA scores was detected in the NPC patients during the longitudinal following (*F* = 23.214, *P* < 0.001; Figure 2); furthermore, there were significant differences in the MoCA scores between pre-RT and EDS (*P* = 0.003), between pre-RT and LDS (*P* < 0.001), and between EDS and LDS (*P* < 0.001) (Figure 2). In the controls, no significant difference was detected in the MoCA scores among the three time points (*P* = 0.257). Ten NPC patients with post-RT NPC at LDS had cognitive function impairment.

### Longitudinal ReHo Changes During Three Periods

**Figure 3:**
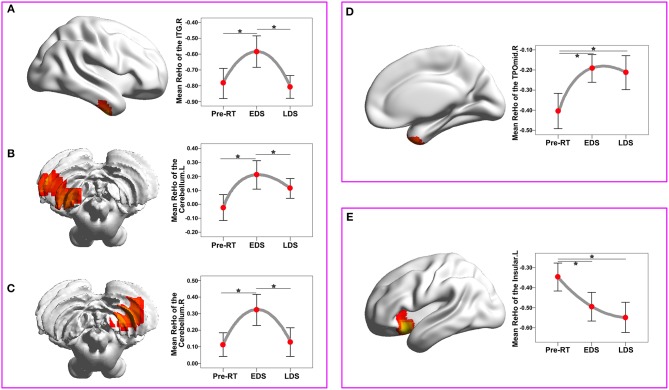
ReHo changes among the three time points compared with a whole-brain voxel-wise based one-way repeated measure ANOVA and *post-hoc* tests. There were three patterns of dynamic change for the ReHo index: “increase-decrease-recover” pattern **(A–C)**, with full-recovery in the right cerebellum **(A)** and right ITGs **(B)**, and with partial-recovery in the left cerebellum **(C)**; “increase” pattern without significant recovery in the right TPOmid **(D)**; “decrease” pattern without significant recovery in the left insula **(E)**. Brain regions were reported at the significant level of a threshold of two-tailed voxel-wise *P* < 0.01 and cluster level *P* < 0.05 with Gaussian Random Field correction. Error bars indicate two standard errors, and pentagrams indicate significant differences revealed by ANOVA *post-hoc* tests with Dunnett's tests (*P* < 0.05). ANOVA, analysis of variance; EDS, early-delayed stage; L, left; LDS, late-delayed stage; ITG, inferior temporal gyrus; MNI, Montreal Neurological Institute; R, right; ReHo, regional homogeneity; TPOmid, temporal pole: middle temporal gyrus; RT, radiotherapy.

The ReHo changes among three periods in patients with NPC are shown in Figure 3 and Table 3. There was a significant difference in the bilateral cerebellum, the right inferior temporal gyrus (ITG), the right temporal pole: the middle temporal gyrus (TPOmid) and the left insula. In healthy controls, there were no significant ReHo changes in any brain region among the three time points.

**Table 3:** Regions of the brain in which the ReHo differ significantly among the pre-RT, EDS, and LDS in the patients with nasopharyngeal carcinoma post RT.

**Brain areas**	**No. of voxels**	**BA**	**MNI coordinate**			**Mean ReHo value**			**Peak *t*-values**
			***X***	***Y***	***Z***	**Pre-RT**	**EDS**	**LDS**	
Right cerebellum	103	-	24	−60	−54	0.113 ± 0.201	0.322 ± 0.265	0.128 ± 0.244	9.390
Left cerebellum	57	-	−48	−66	−54	−0.024 ± 0.261	0.210 ± 0.287	0.113 ± 0.199	13.067
Right ITG	64	20	42	−9	−39	−0.404 ± 0.247	−0.192 ± 0.194	−0.213 ± 0.238	12.236
Right TPOmid	85	38	42	18	−36	−0.785 ± 0.268	−0.584 ± 0.280	−0.807 ± 0.203	8.978
Left insula	71	47	−30	15	−12	−0.347 ± 0.197	−0.495 ± 0.202	−0.548 ± 0.212	12.3708

*BA, Broadmann Areas; EDS, early-delayed stage; LDS, late-delayed stage; ITG, inferior temporal gyrus; MNI, Montreal Neurological Institute; ReHo, regional homogeneity; TPOmid, temporal pole: middle temporal gyrus; RT, radiotherapy*.

The dynamic change curves of mean ReHo extracted from each brain region in patients with NPC across the three stages are depicted in Figure 3. In detail, there were three patterns of dynamic change for the ReHo index: “increase-decrease-recover” pattern in the right cerebellum, the right ITGs and the left cerebellum; “increase without recovery” pattern in the right TPOmid; “decrease without recovery” pattern in the left insula.

### Prediction of Impaired Cognitive Function in LDS Period Using the ReHo Index in EDS

**Figure 4:**
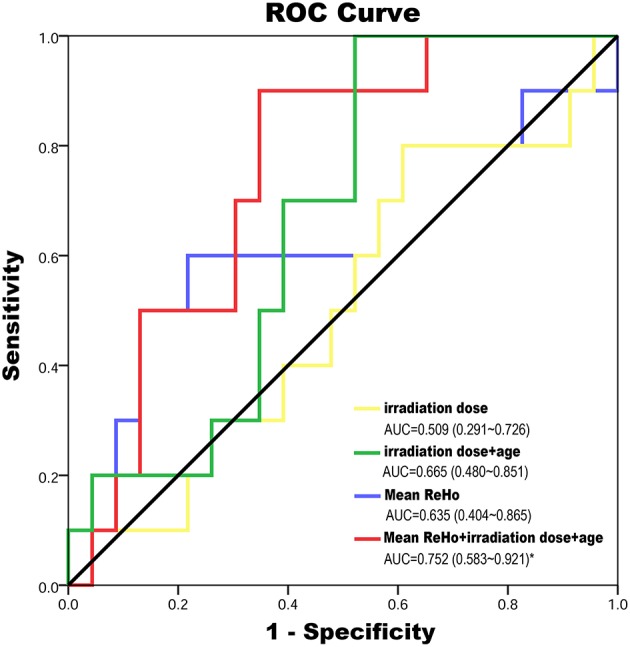
The ROC curves for mean ReHo (showed groups differences) at EDS, age, dose (the maximum irradiation dose to bilateral temporal lobe) alone and three combined model were qualified to determine the efficacies of predicting of impaired cognitive function in LDS. The multivariate regression analysis showed that the logistic regression model combining the three variables has the highest diagnostic efficiency according to the AUC score of the ROC curves (AUC = 0.752, *P* = 0.023). AUC, area under the curve; EDS, early-delayed stage; LDS, late-delayed stage; ReHo, regional homogeneity; ROC, receiver operating characteristic curve; RT, radiotherapy.

According to the established logistic regression models, the ROC curves for mean ReHo (showed groups differences) at EDS, age, dose (the irradiation dose to bilateral temporal lobe) alone, and three combined models were qualified to determine the efficacies of predicting the impaired cognitive function in LDS (Figure 4; Supplementary Table 1). The multivariate regression analysis showed that the logistic regression model combining the three variables had the highest diagnostic efficiency based on the area under the curve (AUC) score of the ROC curves (AUC = 0.752, *P* = 0.023).

## Discussion

To the best of our knowledge, this is the first study to dynamically and longitudinally explore the RT-related aberrant spontaneous brain activity from EDS to LDS in NPC patients. We found that the brain regions showing aberrant ReHo were located in the right ITG, the right TPOmid, the bilateral cerebellum, and the left insula in post-RT NPC patients. Furthermore, these regions showed different patterns of dynamic changes of ReHo: “increase-decrease-recover,” “increase pattern,” and “decrease pattern.” Moreover, the logistic regression model combining the mean ReHo at EDS, age and irradiation dose had the highest efficiency to predict cognitive dysfunction at LDS in patients with NPC. Identification of the aberrant and dynamic ReHo changes in multiple brain regions would contribute to obtaining a better understanding of the characteristics of functional evolutional processes during different phase post RT in NPC patients. Importantly, the combination of the ReHo alterations at EDS, age, and irradiation dose may serve as a potential biomarker for the RT-induced brain functional impairments at LDS.

RT-induced brain injury in the temporal lobe and cerebellum has been well-documented in previous studies (12, 27–30), and these findings are not surprising, given that anatomically they are all close to or overlapped by the CTV, which may cause unnecessary radiation and results in the radioactive damage. In the present study, we observed that the abnormal ReHo in the bilateral cerebellum and the right ITG manifested dynamic changes with an “increase-decrease-recover” pattern. The blood-oxygen level dependent (BOLD) signal is mostly contributed by cerebral blood flow (CBF), and large CBF change has been postulated to produce large BOLD signal variability (31). Thus, abnormal RT-related ReHo observed in present study may also be due to the aberrant RT-induced brain CBF, which in turn relate to the RT-related vascular injury. It is well-known that radiation has profound time-dependent effects on the vasculature (8, 32, 33). Shortly after RT, vascular structure and function can be altered, including blood vessel dilatation, endothelial cell enlargement, increased vascularity, which can lead to acute blood brain barrier (BBB) disruption and increased permeability (34, 35). Then, these acute post-RT alterations were reported to be followed by a recovery process, such as full or partial recovery of the endothelial density, vessel density, and vessel length, though the duration and extent of this recovery seemed to vary among the reports (8, 32). Intriguingly, the present finding that the “increase-decrease-recover” pattern of ReHo in the bilateral cerebellum and right ITG was coincident with the dynamic time course of the vascular post-RT damage, further confirmed the hypothesis that the aberrant ReHo in these regions were associated with vascular injury. Moreover, this hypothesis was also highly supported by the findings of a recent study, through measuring CBF by arterial spin labeling (ASL)-MRI, Hu et al. revealed that elevated CBF in the left cerebellum in post-RT NPC patients at EDS, which recovered to the baseline level at the LDS (28). Additionally, this dynamic pattern was also consistent with other *in vivo* studies by using diverse MRI models, reporting increased fALFF in the ITG (13), as well as decreased diffusion tensor imaging (DTI) and magnetic resonance spectroscopy (MRS) metrics in the temporal lobe at the early stage but partially recovered later (36, 37). Taken together, it is reasonable to conclude that findings of “increase-decrease-recover” change pattern of ReHo in the bilateral cerebellum and the right ITG might be associated with the dynamic interaction between RT-induced vascular disruption and recovery processes.

Notably, we also found abnormal ReHo in the right TPOmid, which showed an “increase without recovery” pattern, whereas a “decrease without recovery” pattern was established in the left insula. Although the underlying biological mechanisms of these diverse patterns are unclear, the dose of the irradiation may explain these phenomena. The dose-volume effects on the brain have been consistently studied previously (23, 29, 38). The temporal pole, which covers the anterior-most end of the temporal lobe, is the brain region that receives the highest dose of radiation. This relatively high dose may lead to a sustained injury without recovery.

Nevertheless, the left insula, which is not exposed to the high dose of RT, showed a “decrease without recovery” pattern; the decreased ReHo in these areas may be interpreted as indirect RT-induced injury. Of note, the insula not only integrates the multimodal sensory information due to the presence of dense connections with other brain regions, such as the frontal and temporal ones, but is also involved in the multiple resting-state networks (12, 39, 40). Recently, we found that the changes of the right insular FC correlated with the maximum dose in the right temporal lobe (12), which indicated that the FC impairment in the right insular may be related to the RT-induced injury in the right temporal lobe. Therefore, it is possible that the decreased ReHo in the left insula could be a secondary RT-induced change caused through the formation of abnormal connections between the insula and a certain vulnerable region (such as the temporal lobe). The finding that a sustained reduced ReHo in NPC patients at both EDS and LDS post RT was supported by previous resting-state fMRI studies. In post-RT NPC patients, not only significantly reduced FC was detected in the right insular within the salience network in NPC patients at the EDS (3 months post-RT) (12), but also aberrant FC related to insula at the LDS (from 6 to 87 months post-RT) (11), However, further studies are needed to elucidate the underlying biological mechanisms.

Interesting, that the combination of brain activity at EDS, irradiation dose and age can be used to predict cognitive impairment at LDS in NPC patients. Given that cognitive impairment is considered to be progressive and irreversible during the LDS (4, 33, 41), the early indications at the reversible stage would be extremely helpful. Our finding was in line with the increasing evidence from recent studies using other MRI modalities, such as dynamic contrast-enhanced (DCE) MRI and DTI (7, 38), which showed that the early hippocampal vascular dose response and the early diffusivity changes in the parahippocampal cingulum can be useful as a biomarker for predicting late-delayed cognitive decline. Our findings also supported the hypothesis that the process of RT-induced cognitive function impairment at LDS is multifactorial (4, 7, 38, 42, 43). Irradiation dose-dependent brain cognitive function changes have been well-documented in previous studies (4, 43). Recently, increasing evidence reveals that radiation-induced cognitive dysfunction is significantly influenced by age (38, 42). Thus, our findings added to the current literature that the RT-induced cognitive impairment is a multifactorial influenced process, and the combination of the ReHo alterations at EDS, age, and irradiation dose may serve as a potential biomarker of the RT-induced brain cognitive impairments at LDS.

Nevertheless, still some limitations in this study should be mentioned. First, the MoCA used in the present study is a brief cognitive screening tool not highly sensitive to certain domains (26), such as the verbal and visual memory, attention and executive functions, impaired by RT as documented in previous studies (7, 33). Further studies with a complex cognitive evaluation are warranted to focus on the relationship between specific cognitive impairments and abnormal ReHo to fully assess the effect of RT-induced early brain activity changes on late-delayed cognitive decline in NPC patients. Second, in the current study, we longitudinally observed the alterations of the spontaneous brain activity and the general cognition within 6 months post RT in NPC patients. Thus, whether these phenomena were permanent or transient is still an open question, and future studies with longer follow-up periods are needed to obtain a better understanding of the characteristics of the processes of functional changes. Third, in this preliminary study, the diagnostic performances of the model to predict the cognitive late-delayed impairments after RT was moderate (AUC = 0.752), with a relative high specificity (90%) but a low sensitivity (65.2%). A more comprehensive models incorporating clinical and multiple parameters derived from multimodal MRI is warranted to improve the diagnostic efficiency in the future. Finally, another limitation could be a potential learning effect of neurocognitive testing due to repeated use (three times in 6 months) of the MoCA test. This could be reduced or avoided by using parallel versions when possible and by a well-structured time scheduling in the future study (44).

## Conclusion

Longitudinal analyses of brain activity changes provide new important insights into radiation-induced brain functional impairments in NPC patients after RT. We found that aberrant ReHo was mainly distributed in the temporal lobe and the cerebellum, which received a comparatively higher dose of irradiation. Furthermore, alterations of ReHo in these brain regions manifested as different patterns over time, which revealed that the pathophysiology of post-RT brain injury is dynamic, complex, and multifactorial. More importantly, the combination of the ReHo alterations at EDS, age, and irradiation dose may serve as a potential biomarker for the cognitive late-delayed impairments after RT. Therefore, our preliminary findings will provide guidance for critically important early interventions before permanent and irreversible disability has occurred at LDS.

## Ethics Statement

This study was carried out in accordance with the recommendations of name of guidelines, name of committee with written informed consent from all subjects. All subjects gave written informed consent in accordance with the Declaration of Helsinki. The protocol was approved by the Sun Yat-sen University Cancer Center.

## Author Contributions

YY, XLv, and YQ designed of the study and carried out data collection and wrote the manuscript. XLin screened the clinical data of the study. JL and LH completed the acquisition of functional imaging data. ZL and SL sorted the data. YQ carried out data analysis. CX and GH contributed to conceptualization of the study and revision of the manuscript.

### Conflict of Interest Statement

The authors declare that the research was conducted in the absence of any commercial or financial relationships that could be construed as a potential conflict of interest.
