# Supplementary material for: Aberrant Brain Activity at Early Delay Stage Post-radiotherapy as a Biomarker for Predicting Neurocognitive Dysfunction Late-Delayed in Patients With Nasopharyngeal Carcinoma
Source: Front Neurol. 2019 Jul 16;10:752. doi: 10.3389/fneur.2019.00752 (PMC6660255; doi:10.3389/fneur.2019.00752)
Supplement: Supplementary file 1 [file Table_1.DOCX]

Supplementary Material

**Supplementary Table 1.** ROC analysis for determine the efficacies of the mean ReHo value within all the clusters that showed significant changes among three time points at EDS and clinical characteristics alone or combined to predict impaired cognitive function at LDS.

| Characteristics | Cut off | AUC (95%CI) | Sensitivity(%) | Specificity(%) | PPV (%) | NPV (%) | P-value |
| --- | --- | --- | --- | --- | --- | --- | --- |
| Irradiation dose | 65.9Gy | 0.509 | 80% | 39.1% | 36.4% | 81.8% | 0.509 |
| Irradiation Dose*+Age | - | 0.665 | 100% | 47.9% | 45.4% | 100% | 0.137 |
| Mean ReHo value | -0.0743 | 0.635 | 60% | 78.3% | 54.5% | 81.8% | 0.225 |
| Mean ReHo value+ Irradiation Dose*+Age | - | 0.752 | 90% | 65.2% | 52.9% | 93.8% | 0.023 |

* The maximum irradiation dose of bilateral temporal lobe. AUC, area under the curve; EDS, early delayed stage; LDS, late delayed stage; ReHo, regional homogeneity; ROC, receiver operating characteristic curve.
